# Source of Stress-Associated Factors Among Medical and Nursing Students: A Cross-Sectional Study

**DOI:** 10.1155/jonm/9928649

**Published:** 2025-07-17

**Authors:** Hanif Ullah, Safia Arbab, Chang-qing Liu, Qiujing Du, Sher Alam Khan, Sawar Khan, Omer Dad, Yali Tian, Ka Li

**Affiliations:** ^1^Medicine and Engineering Interdisciplinary Research Laboratory of Nursing & Materials/Nursing Key Laboratory of Sichuan Province, West China Hospital, Sichuan University/West China School of Nursing, Sichuan University, Chengdu 610041, Sichuan, China; ^2^Department of Paediatrics, Musharaf Medical Complex Abbottabad, Abbottabad, Pakistan; ^3^Department of Cell Biology, School of Life Sciences, Central South University, Changsha, China; ^4^Department of Zoology, Hazara University, Manshera, Pakistan

**Keywords:** medical students, mental health, nursing students, Pakistan, stress, stress factors

## Abstract

**Background:** The demanding requirements of nursing education impose great stress on students, which can adversely affect their health and well-being in terms of academic and clinical performance.

**Aims:** This study aimed to determine the level of stress and the main causes of stress among medical and nursing students.

**Methods:** This cross-sectional study was conducted with medical and nursing students in Pakistan via a convenience sampling method. Sources of stress-associated factors were assessed using online questionnaire. The data were collected between July 1 and August 31, 2024. A total of 302 medical and nursing students participated in the research. Descriptive statistics (frequency, mean) and inferential statistics were used to analyze the collected data.

**Results:** According to the findings, medical and nursing students experience stress from multiple sources, with financial difficulties, academic workload, clinical practice, and environmental factors being the primary contributors. The most common stressors were insufficient income, pressure from clinical instructors and staff, and unfamiliarity with patient diagnoses and treatments. Multifactor logistic regression analysis revealed that stress from assignments and workload was significantly associated with fear of poor grades (*p*=0.046), while clinical practice quality also showed a significant effect (*p*=0.029). Additionally, dealing with patients with physio-psychosocial issues (*p*=0.039) and unfamiliarity with clinical conditions (*p*=0.012) were significant predictors of stress. These findings highlight the complex and multifaceted nature of student stress.

**Conclusion:** This study revealed that medical and nursing students experience significant levels of stress related to finances, clinical practice, and academics, which affects their performance.

**Medical and Nursing Implications:** Educational administrators must establish policies that assist medical and nursing students. Some of these policies could include evaluating the workload of Medical and nursing students, refining curriculum design, providing financial support, and providing scholarships to students facing challenging circumstances.

## 1. Introduction

Every individual experiences stress, which has an extensive impact on mental health as well as physical health and well-being. Stress is a consequence of a dynamic interplay between a person's surroundings and themselves. The demands, constraints, and possibilities associated with the task may be seen in this interaction as posing a risk to an individual's abilities and resources [1, 2]. Stress can impair focus, memory, and problem-solving skills, all of which harm learning and academic achievement, making it a particularly significant issue in education [3, 4]. Because of the intense curriculum, clinical rotations, and patient care responsibilities, nursing students frequently experience significant levels of stress and academic pressure. These pressures can negatively impact nursing students' quality of life, which can therefore have an adverse effect on their physical and mental health as well as their general well-being. Research indicates that among nursing students, stress and academic pressure can result in burnout, anxiety, depression, and other mental health problems [5, 6].

Studies reported worldwide have revealed that the nursing curriculum is one of the most demanding academic programs, resulting in detrimental effects on the physical and emotional well-being of nursing students [7]. Students in nursing face similar stressors as their counterparts in other academic programs but also confront pressures unique to the nursing curriculum's practical training component of the nursing curriculum [8, 9]. To effectively complete their nursing courses, students must possess a strong sense of commitment, dedication, and a willingness to meet additional time demands. One of the greatest stressors that can lead to burnout and diminished well-being is the persistent expectation that students demonstrate a high level of knowledge and skill in both academic and clinical settings as they develop into competent, knowledgeable registered nurses [10]. Stressors such as academic pressure, clinical workload, financial issues, and emotional demands significantly impact nursing students by increasing anxiety and burnout while reducing academic performance. These challenges can negatively affect mental well-being, impede skill acquisition, and compromise patient care during training. Effective stress management is crucial for their success and health [11]. The mental and physical well-being of individuals may be adversely impacted by work-related stress, which is one of many possible stress sources. High levels of stress have also been linked to low productivity and high employee absenteeism [12]. Another stress source is related to therapeutic practice. Musa et al. reported that two of the most common causes of stress were caring for patients and feeling a lack of knowledge and professional skills. Specific reasons associated with these highly stressful events included fear of failing grades, unfamiliarity with medical terminology and background, and a lack of experience and ability to provide nursing care and diagnostics [13]. Academic pressure is also a source of stress. Stressful situations for nursing students include exams and assessments, apprehension about failing courses, and managing financial aid [14].

Studies from Nepal, Egypt, Brazil, Vietnam, and other countries consistently show that nursing and medical students experience significant stress, with varying prevalence rates. In Nepal, 60.4% of nursing students reported moderate stress, whereas studies from Egypt (62.4%), Brazil (64%), and Vietnam (38.5%–47.6%) also highlighted high levels of stress among students [15–18]. Research has also indicated that stress among nursing students can be attributed to four categories of factors: clinical, educational, financial, and confidence. Stress refers to the emotional and physical response to challenges or demands, whereas stressors are the external events or situations that trigger that response. Stressors can be environmental, social, or internal factors. Studies conducted in Saudi Arabia revealed that the clinical practice setting was one of the main causes of stress for nursing students [19, 20]. Stress can be caused in students studying clinical practice in the hospital by factors such as patient attitudes, anxiety about making mistakes, and having to deal with patients' pain or death. Stress is also caused by a variety of educational variables, including the pressure to study, tests, challenging readings, and the fear of failing the course [19, 21]. Several characteristics, such as age, sex, year of study, and learning environment, are associated with the incidence of stress and anxiety among nursing students. The results, however, are mixed. According to some studies, the stress and anxiety of freshmen, females, and younger students are greater [22]. According to other research, male, senior, and older students experience higher levels of psychological distress [23]. However, other research revealed that stress and anxiety did not significantly change according to a person's gender or academic year [24].

It is important for society that students obtain the knowledge and abilities that will enable them to make constructive contributions to the growth of any country's overall economy. However, there are instances where the stress of the academic setting overpowers students, counteracting the benefits that one might anticipate from graduating from university [25, 26]. Early stress management can help reduce its effects and help students become more well-adjusted, according to earlier studies. Therefore, to enable nursing students to take charge of their academic stress and stress, health promotion initiatives, including stress management and healthy coping skills as well as lifestyle changes, are needed [27]. The most common causes of stress include fear of failure, uncertainty about the future, a lack of confidence, uneasiness, anxiety, melancholy, depression, and a lack of self-assurance. Sour disposition, short fuses, lethargy, poor sleep and low performance satisfaction are indicators that are defined as markers or pointers that indicate something, provide direction, and raise awareness of what has to be done [1].

Conducted within the framework of Pakistan's medical and nursing education systems known for their intense academic demands, extended training hours, and emotional burdens this research sheds light on the additional pressures posed by limited access to mental health resources, financial difficulties, and deeply rooted cultural expectations. These compounded stressors create a unique set of challenges that are not fully addressed in existing global literature.

The novelty of this study lies in its focus on identifying culturally and contextually specific sources of stress within the Pakistani context a dimension often overlooked in broader, internationally focused research. By concentrating on local socio-economic and cultural dynamics, this study offers original insights that can help institutions in Pakistan implement more relevant and effective mental health interventions, uniquely tailored to the needs of their students.

The objective of this study was to explore and analyze the specific stress-inducing factors faced by medical and nursing students in Pakistan. By gaining a comprehensive understanding of these stressors, the study seeks to guide the creation of targeted strategies and institutional interventions aimed at reducing stress levels, enhancing mental well-being, and ultimately improving both academic performance and overall quality of life for students.

## 2. Methods

### 2.1. Study Design

The study was a cross-sectional study conducted in Pakistan among nursing and medical students from government and private healthcare centers. The STROBE checklist was used for reporting this study.

### 2.2. Inclusion and Exclusion Criteria

A convenience sampling method was used for medical and nursing students who were available and willing to participate. The eligibility criteria for this study required participants to be enrolled in medical or nursing degree programs offered by the college during the academic year and to voluntarily consent to participate. Students were selected from the second semester of the first year up to the final year of their academic program. At the same time, those who were absent due to sickness or leave of absence during the data collection period were excluded.

### 2.3. Data Collection

A total of 309 questionnaire responses were collected from July 1 to August 31, 2024. A survey was created via Google Forms (https://docs.google.com/forms/), and link was shared via WhatsApp. Only the core members had access to the data repository, ensuring participant privacy. After data collection was completed, the data was exported to Excel for further analysis. The researcher first addressed the goals, significance, and primary subject matter of the study while also responding to questions from the students. Students who consented to participate in this study verified the approved list. The required sample size was calculated from the Raosoft sample size calculation [28–30]. Sample size calculation was performed via Raosoft software based on a population size of 600 and a response distribution of 50%. A margin of error of 5% and a confidence interval of 95% were used. Based on these parameters, the required sample size was 235. However, to obtain more exact and accurate results, we collected 309 responses. After excluding seven incomplete or inconsistent responses, 302 valid questionnaires were included in the final analysis. It was confirmed that no information was missing since all the questions had to be answered; thus, only fully completed questionnaires were collected. The study was voluntary, and the participants were confident that all the information gathered would be kept private and confidential. They could also resign from the study without losing their right to rewards. The students studying nursing and medical schools helped distribute the questionnaires on an individual basis as well as through WhatsApp groups. The questionnaire took 10–15 min to complete.

### 2.4. Measures and Instruments

A questionnaire was developed based on relevant published articles and scientific literature [31–33]. It was administered in English, Pakistan's official language; the questionnaire aimed to collect comprehensive data on the subject. Pretest helps researchers test the data collection procedures and identify any issues before the main study and refine the data collection tools or instruments based on feedback to ensure clarity and relevance. The survey was divided into two sections. First, the research team created a set of sociodemographic questions based on the demographic characteristics of the nursing students, including their academic year, age, gender, marital status, place of residence, number of roommates, amount of time spent studying on their own, and time spent sleeping [31]. The second section utilized the Perceived Stress Scale (PSS), developed by Sheu et al., which comprises 22 items presented on a 5-point Likert scale [34]. These items were categorized into six dimensions: stress related to teachers and staff (4 items), stress from patient care (6 items), stress associated with assignments and workload (6 items), stress arising from the clinical environment (3 items), and stress due to a lack of professional knowledge and skills (3 items) [32, 33].

The total score from the questionnaire serves as the foundation for assessing stress levels, with the following categories: normal (0–14), mild (15–18), moderate (19–25), severe (26–33), and extremely severe (≥ 34). Students are asked to respond based on their personal experiences, selecting from five options for each item, ranging from 1 (normal) to five (extremely severe).

The PSS developed by Sheu et al. is specifically designed to assess clinical stress among nursing and medical students. Its construct validity was confirmed through factor analysis, which identified six dimensions of stress: patient care, teachers and staff, assignments and workload, peer interactions, lack of professional knowledge, and the clinical environment. The scale demonstrated high internal consistency, with a Cronbach's alpha of 0.89, supporting its reliability and validity in assessing stress levels among nursing and medical students. The Content Validity Ratio (CVR) was 0.91, indicating strong expert agreement on the relevance of the items [34].

### 2.5. Statistical Analysis

The data collected from the participants were initially entered into Google Worksheets and then exported to SPSS 17.0 and Microsoft Excel. The quantitative analysis formed the basis for the data analysis, which integrated descriptive and inferential statistics. For every study variable regardless of whether it was an independent or dependent descriptive variable, multiple logistic regression analyses were carried out using frequency counts and percentages.

### 2.6. Ethical Statement

The study was approved by the Ethical Research Committee of Abbottabad Pakistan Kidney Center No: (PKC-2024-0220), Abbottabad. Respondents must utilize the 'informed consent' declaration on the first page to assent (accept or deny) to our online survey.

## 3. Results

### 3.1. Demographic Characteristics of Medical and Nursing Students

A total of 309 questionnaires were collected; 7 were excluded because of missing data, and 302 participants were excluded for the final analysis. As shown in Table 1, the majority of the respondents were male (55.30%), followed by females (44.70%). In terms of academic year, most students were in their fourth year (49.67%), while first-year students represented the smallest group. There was considerable variation in the number of students across different academic years. With respect to education, most participants were in medical programs (66.89%), followed by nursing students (33.11%). According to the study habits, most students dedicated more than 5 h daily to self-study, followed by those who studied for 3-4 h daily. Most of the students lived in college hostels, whereas a smaller proportion lived at home. Concerning sleep patterns, nearly half of the students reported sleeping between 6 and 8 h per night. Financially, the largest group of students reported having insufficient income, followed by those who described their income as barely sufficient based on their expenses and financial needs. The overall findings revealed that most participants were male and in medical programs. Key factors such as study habits, residences, sleep patterns, and financial status varied, influencing their overall academic experience and stress levels, as shown in Table 1.

### 3.2. Stress Levels in Medical and Nursing Students

Stress among medical and nursing students is a significant concern and arises from various factors, such as heavy study loads, academic pressure, and the challenges of transitioning into clinical practice. Among the 302 medical and nursing students, most reported a normal stress level (50.66%), followed by moderate stress (23.18%). A smaller proportion of the students reported mild stress, whereas the lowest percentage reported severe stress, as shown in Table 2. These findings highlight varying degrees of stress among students, emphasizing the need for effective strategies and support systems to help manage academic and clinical challenges.

### 3.3. Multiple Logistic Regression Analyses of Pakistani Medical and Nursing Students' Stress

All continuous variables were skewed (*p* > 0.05), and stress levels were analyzed as categorical variables in multiple logistic regression analyses. In the multifactor logistic regression analysis of variable stress from assignments and workload, the results revealed that students worried about bad grades (*p*=0.046), B and Exp(B) were (−0.041 and 0.959), and pressure from the quality of clinical practice was statistically significant (*p = *0.029), among which B and Exp(B) were (−0.047 and 1.048), respectively. We found that poor grades are negatively associated with stress caused by assignments and workload. The pressure stemming from the quality of clinical practice is positively associated with stress caused by assignments and workload and pressure from the impact of the quality of clinical practice on stress from assignments and workload is more significant than worry about bad grades.

In the multifactor logistic regression analysis of stress among medical and nursing students due to a lack of professional knowledge and skills, the results indicated that being unfamiliar with patients' diagnoses and treatments was statistically significant (*p* = 0.012), with B and Exp(B) values of (−0.049 and 0.953), respectively. This finding suggests that low familiarity with diagnoses and treatments is associated with increased stress levels among students. However, stress from a lack of professional knowledge and skills are negatively associated with being unfamiliar with patients' diagnoses and treatments.

The results of the multifactor logistic regression analysis of stress from taking care of patients revealed that worry about not being trusted or accepted was statistically significant (*p*=0.012), with B and Exp(B) values of 0.056 and 1.058, respectively. Additionally, not knowing how to help patients with physio-psychosocial problems was also significant (*p*=0.039), with B and Exp(B) values of (−0.049 and 0.952), respectively. The value of worry about not being trusted or accepted suggests that this concern is associated with increased stress levels. In contrast, the negative value for uncertainty in addressing patients' physio-psychosocial issues indicates an inverse relationship, implying that greater confidence in managing these problems may reduce stress. The detailed outcomes are presented in Table 3.

## 4. Discussion

Nursing and medical students are regularly subjected to stressors during their education and training, which may directly or indirectly impair their performance and ability to learn. The demands of practical teaching involve difficulties that could stress students. The curriculum is more stressful than other programs because of its practical components, which are important in preparing students for the development of a professional nursing role [14].

The results of our study revealed that different factors contribute to stress; academic, clinical, and environmental challenges are the main sources of stress for nursing and medical students. Overall, 23.18% of the students reported experiencing moderate levels of stress. Similarly, the findings of this study are consistently reported by other researchers, the rate of moderate stress observed here is lower than that reported in studies of nursing and medical students in the United States, Hong Kong, India, Vietnam, and Nepal. In those studies, moderate stress was reported at rates of 38%, 32.56%, 27%, 20%, and 33.9%, respectively [21, 25, 35–37]. Furthermore, studies on stress among Egyptian medical students (62.4%) and students from Vietnamese National University (68.29%) reported considerably higher levels of stress [16, 38].

Our findings also demonstrated that the factor, including financial (38.10%) and clinical aspects, was linked to the stress levels of medical and nursing students. Financial stress among Pakistani students is often caused by the high cost of education, including tuition fees and other expenses. Many students also face financial pressures from their families, which can lead to anxiety about meeting educational and personal needs. This finding demonstrates that one significant factor influencing students' stress levels is finance. Similarly, the results are consistent with those of Cheung et al. [21]. This outcome differs from research conducted with nursing students in Macao and Malaysia [39, 40].

The results of our study demonstrated that clinical stress (*p*=0.029) was the primary source of stress for nursing and medical students. Similarly, this finding is supported by research from Saudi Arabia, which revealed that one of the main sources of stress for nursing students is their clinical practice [19]. According to a Hong Kong study, nursing students' stress levels are correlated with clinical pressure [21]. Most of the parameters related to confidence increased the risk of stress among nursing and medical students, according to this study. Since many students are far from their families, friends, and teachers, this has a large effect on students' mental health. Furthermore, stress is also more likely to occur when personal issues arise. This outcome is consistent with research conducted by students at Thang Long University and Tien Giang Medical College [41].

The present study revealed that providing care to patients (*p*=0.012) and concerns about not being trusted or accepted by patients and their families were the most significant sources of stress for students. Similarly, reported stressors often lead to emotional challenges, including feelings of embarrassment or belittlement, as described by Al-Qerem et al. [42]. These findings align with earlier research by Khater et al. [43], who reported similar stressors among students in clinical practice settings. The pressure to meet patient expectations and establish trust can exacerbate these feelings, further impacting students' confidence and performance. Addressing these issues is crucial for fostering a supportive learning environment that mitigates stress and enhances student well-being.

Medical and nursing students face rigorous academic and clinical demands, with the nursing curriculum playing the most significant role in shaping their training. In addition, the submission of assignments, homework, assessment deadlines, projects, reports, term papers, quizzes, and exams makes the program more rigorous or demanding to the point that it is more than other programs [44, 45]. The present study revealed that fewer than three-quarters of the students experienced significant levels of academic stress from their workload and tasks. Similar results were reported by Yusoff et al., who reported that nursing students believe that their clinical assignments, particularly nursing care plans, are extremely demanding [46, 47]. The study revealed that the students reported academic stress because of their deficiency in professional knowledge and skills. Capdevila-Gaudens et al. and Khater et al. reported the same results: a second source of academic stress, of the medical and nursing students' perceived lack of professional knowledge and ability, and students' perceived lack of professional knowledge and skills, particularly junior students, was the second source of academic stress [43, 48].

In Western settings where stress may often stem from competitive learning environments or personal autonomy in career choices, many Pakistani students face challenges, intense family expectations, and societal pressure to pursue medicine or nursing, often as a means of social mobility. Similar patterns have been observed in South Asian countries, such as those conducted in India and Bangladesh, where parental expectations and financial concerns significantly impact students' stress levels [49, 50].

One significant challenge in Pakistan is the socio-cultural expectation placed on students, particularly those in the medical and nursing professions. In many Pakistani families, choosing medicine or nursing is often not a personal choice, but a decision influenced by parental expectations and societal status [51]. Another challenge is the limited availability of structured student support services, such as counseling, stress management workshops, or mental health interventions. Unlike universities in developed countries where student wellness programs are well-established, Pakistani institutions often lack the necessary infrastructure to support student well-being [52]. This deficiency makes it harder for students to seek help, leading to unaddressed stress that may affect their academic performance and overall well-being.

The clinical learning environment in Pakistan also poses challenges. Medical and Nursing students frequently experience understaffed hospitals, resource scarcity, and emotionally challenging patient interactions with little to no mentorship. In contrast to countries where students are gradually introduced to clinical settings under close supervision, Pakistani students often face abrupt and intense exposure [53]. This abrupt transition can lead to anxiety, self-doubt, and emotional fatigue.

### 4.1. Medical and Nursing Implications

Our research provides valuable insights for clinical staff members and educators, helping them better understand the complexities of students' stress responses. This emphasizes the importance of adapting traditional approaches when interacting with students in clinical settings. Additionally, allowing students to spend more time in a single ward during their clinical rotations, rather than rotating through multiple wards for short periods, will help them become more comfortable with the environment and establish stronger connections with staff and patients. This continuity will enable students to gain a deeper understanding of clinical practices. Providing training courses as stand-alone units with specific tasks to be completed within designated working hours would also help students develop professional nursing skills. This approach would create an environment where students can engage in meaningful practice while preparing for their future careers as competent and independent healthcare professionals. The findings highlight the need for targeted stress management programs tailored to the unique challenges faced by medical and nursing students. Institutions should prioritize mental health support, promote a balanced academic workload, and foster a supportive learning environment to enhance student well-being and academic performance.

### 4.2. Limitations of the Study

This study has several limitations that may have influenced the findings. Firstly, the use of a self-report questionnaire introduces the potential for reporting bias, as participants may interpret and respond to questions differently based on their perceptions. This convenience sampling method, which carries several limitations, it may lead to sampling bias, as participants are not randomly selected, potentially skewing the results. Additionally, the sample may not accurately represent the broader population, limiting the generalizability of the findings to all medical and nursing students. Secondly, while general stress levels were assessed, stress specifically associated with clinical settings, an important contributor to student stress, was not explored, leaving a gap in understanding clinical stressors. Additionally, the study's cross-sectional design only provides a snapshot of stress at a single point in time, making it difficult to capture fluctuations in stress levels due to varying academic or personal circumstances. This limits the ability to identify long-term patterns or trends in stress. A longitudinal study would be more effective in tracking how stress evolves. Future research should also focus on evaluating the effectiveness of coping strategies, interventions, and institutional support systems in reducing stress and improving student well-being and academic performance.

## 5. Conclusion and Recommendations

In conclusion, this study identified key sources of stress among medical and nursing students, with clinical and environmental factors being the most significant contributors. The findings suggest that stress levels ranging from moderate to high are driven by academic workload, clinical training, and financial constraints. Among these, clinical practice presents the greatest challenge, as students must navigate unfamiliar environments, high expectations, and complex patient interactions. To address this issue, educators and clinical mentors should implement strategies to support students, alleviate stress, and create a more positive learning experience. Institutions should provide accessible mental health support, including on-campus counseling services, peer support programs, and routine stress management workshops. Revising academic curricula to promote a more balanced workload and alleviate excessive academic pressure is also essential. Incorporating stress management education into the curriculum can help students build effective coping mechanisms.

## Figures and Tables

**Table 1:** Demographic characteristics of 302 medical and nursing students.

Variable	*N* (%)
*Gender*	
Female	135 (44.70)
Male	167 (55.30)

*Marital status*	
Married	102 (33.78)
Single	200 (66.22)

*Age*	
< 20	38 (12.60)
20–25	138 (45.70)
> 25	126 (41.72)

*Year of study*	
First year	29 (9.60)
Second year	44 (14.57)
Third year	79 (26.16)
Fourth year	150 (49.67)

*Accommodation*	
Hostel	167 (55.30)
Relative's house	59 (19.56)
Your house	76 (25.17)

*Collage type*	
Government	147 (48.68)
Private	155 (51.32)

*Education*	
Medical	202 (66.89)
Nursing	100 (33.11)

*Income*	
Barely sufficient	100 (33.11)
Insufficient	115 (38.10)
Sufficient	85 (28.15)

*Study time*	
2 h or less	49 (16.23)
3–4 h	102 (33.78)
5 h or more	151 (49.67)

*Sleep time*	
6–8 h	147 (48.68)
Less than 6 h	53 (17.55)
More than 8 h	102 (33.78)

**Table 2:** Rates of stress levels among medical and nursing students.

Stress level	*N* (%)
Extremely severe	5 (1.65)
Severe	26 (8.60)
Moderate	70 (23.18)
Mild	48 (15.90)
Normal	153 (50.66)

**Table 3:** Multiple logistic regression analyses of Pakistani medical and nursing students' stress (*N* = 302).

Variable	B	S.E.	Wald	*p* value	Exp(B)	95.0% C.I. for EXP(B)	
						Lower	Upper
*Stress from assignments and workload*							
Worry about bad grades	−0.041	0.021	3.987	0.046	0.959	0.921	0.999
Pressure from the amount of classwork material to be learned	0.023	0.023	0.991	0.319	1.023	0.978	1.070
Fear from examination	0.010	0.020	0.260	0.610	1.010	0.972	1.049
Teachers feel that performance does not meet.	−0.033	0.021	2.517	0.113	0.967	0.928	1.008
The pressure of clinical practice quality	0.047	0.021	4.762	0.029	1.048	1.005	1.093
Feelings that demand of clinical practice exceed physical and emotional endurance	−0.026	0.020	1.703	0.192	0.975	0.938	1.013

*Stress from teachers and staff*							
Teachers didn't care or lack of guide.	−0.012	0.018	0.475	0.491	0.988	0.954	1.023
Feelings of stress when teachers' instruction is unclear	0.008	0.021	0.141	0.707	1.008	0.968	1.050
Experience the theory practice nucleus	0.017	0.022	0.554	0.457	1.017	0.973	1.062
Cannot discuss patients' situation with teachers	0.001	0.019	0.001	0.978	1.001	0.964	1.038

*Stress from lack of professional knowledge and skills*							
Unfamiliar with professional medical skills	0.028	0.017	2.747	0.097	1.028	0.995	1.063
Unfamiliar with patients' diagnoses and treatments	−0.049	0.019	6.337	0.012	0.953	0.917	0.989
Unfamiliar with medical history and terms	0.032	0.017	3.743	0.053	1.033	1.000	1.067

*Stress from taking care of the patient*							
Patients were unable to get good care	0.004	0.019	0.038	0.845	1.004	0.967	1.042
Doesn't know how to communicate with patients.	0.033	0.020	2.728	0.099	1.033	0.994	1.074
The lack of experience both in making judgments and providing care.	0.011	0.023	0.229	0.632	1.011	0.966	1.059
Do not know how to help the patients with physio-psycho-social problems.	−0.049	0.024	4.240	0.039	0.952	0.909	0.998
Worried about not being trusted or accepted, by patients and families.	0.056	0.022	6.290	0.012	1.058	1.012	1.105
Unable to give doctors, teachers, and patients right answers to their questions	−0.011	0.023	0.238	0.626	0.989	0.946	1.034

*Stress from the clinical environment*							
Unfamiliarity with ward facilities	−0.007	0.020	0.129	0.719	0.993	0.955	1.032
Fear of criticism from staff, teachers	0.038	0.021	3.317	0.069	1.039	0.997	1.082
Lack of empathy and willingness to help, medical personnel.	0.018	0.017	1.097	0.295	1.018	0.984	1.053

## Data Availability

The data that support the findings of this study are available on request from the corresponding authors. The data are not publicly available due to privacy or ethical restrictions.
